# Thrombomodulin as a New Marker of Endothelial Dysfunction in Chronic Kidney Disease in Children

**DOI:** 10.1155/2018/1619293

**Published:** 2018-02-28

**Authors:** Dorota Drożdż, Monika Łątka, Tomasz Drożdż, Krystyna Sztefko, Przemko Kwinta

**Affiliations:** ^1^Department of Pediatric Nephrology and Hypertension, Jagiellonian University Medical College, 265 Wielicka St., 30-663 Krakow, Poland; ^2^1st Department of Cardiology, Interventional Electrocardiology and Hypertension, Jagiellonian University Medical College, 17 Kopernika St., 31-501 Krakow, Poland; ^3^Department of Clinical Biochemistry, Jagiellonian University Medical College, 265 Wielicka St., 30-663 Krakow, Poland; ^4^Department of Pediatrics, Jagiellonian University Medical College, 265 Wielicka St., 30-663 Krakow, Poland

## Abstract

Endothelial dysfunction (ED) and oxidative stress are potential new pathomechanisms of cardiovascular diseases in patients with chronic kidney disease (CKD). The aim of the study was to assess the association between endothelial dysfunction, oxidative stress biomarkers, and cardiovascular risk factors in children with CKD. Serum oxidized LDL (oxLDL), protein carbonyl group, urea, creatinine, cystatin C, thrombomodulin, asymmetric dimethylarginine (ADMA), von Willebrand factor, brain natriuretic peptide (BNP), lipids, high sensitivity C-reactive protein, intercellular adhesion molecule-1 levels, and albuminuria were measured. Anthropometric, ambulatory blood pressure (BP) measurements and echocardiography were performed. The studied group consisted of 59 patients aged 0.7–18.6 (mean 11.1) years with stages 1 to 5 CKD. Thrombomodulin strongly correlated with creatinine (*R* = 0.666; *p* < 0.001), cystatin C (*R* = 0.738; *p* < 0.001), BNP (*R* = 0.406; *p* = 0.001), ADMA (*R* = 0.353; *p* = 0.01), oxLDL (*R* = 0.340; *p* = 0.009), 24-hour systolic (*R* = 0.345; *p* = 0.011) and mean (*R* = 0.315; *p* < 0.05) BP values, and left ventricular mass index (LVMI, *R* = 0.293; *p* = 0.024) and negatively with estimated glomerular filtration rate (*R* = −0.716; *p* < 0.001). In children with CKD, TM strongly depended on kidney function parameters, oxLDL levels, and 24-hour systolic and mean BP values. Thrombomodulin seems to be a valuable marker of ED in CKD patients, correlating with CKD stage as well as oxidative stress, BP values, and LVMI.

## 1. Introduction

Cardiovascular diseases are the most frequent cause of morbidity and mortality in patients with chronic kidney disease (CKD). The adult population with impaired renal function is characterized by high incidence of classical cardiovascular risk factors; however, this cannot explain the high prevalence of cardiovascular events in children with kidney function impairment. Therefore, other factors such as endothelial dysfunction (ED) and oxidative stress are considered. Initially, ED was studied in the most advanced stage of CKD (dialyzed patients) and a reduction in endothelial-dependent vasodilatation of the brachial artery was demonstrated. Recent studies supported the concept that ED is present at early stages of chronic kidney disease [1, 2].

Endothelial cells play a key role in maintaining vascular homeostasis. They regulate vasodilatation, fibrinolysis, and thrombosis through the synthesis and release of substances such as nitric oxide, prostacyclin, thrombomodulin, and tissue plasminogen activator [3, 4]. Endothelial damage results in reduced release of protective substances and at the same time increased release of counteractive substances—endothelin I, angiotensin II, plasminogen activator inhibitor-1 (PAI-1), and von Willebrand factor (vWF), leading to vasoconstriction, platelet activation, and prothrombotic and antifibrinolytic activity.

Noninvasive methods for ED assessment include evaluation of shed endothelial surface layer components—soluble thrombomodulin (TM) and syndecan-1 [5]. TM is a transmembrane glycoprotein built from 5 domains. Its anticoagulant effect is mediated by the binding of thrombin and activation of protein C. Active protein C inhibits the coagulation cascade through degradation of active factors V and VIII and inactivation of tissue plasminogen activator inhibitor. Thrombomodulin has also anti-inflammatory activity. In the case of endothelial cell damage, its transmembrane portion is released and may be identified as a soluble TM (sTM) and the TM molecule loses its vasoprotective properties [6].

Asymmetric dimethylarginine (ADMA), an endogenous inhibitor of nitric oxide synthetase, is the recognized mediator of endothelial injury [7]. Intravenous infusion of small doses of ADMA resulted in an increase of systemic vascular resistance and mean blood pressure in healthy volunteers [8]. Clinical trials have shown that ADMA is not only a risk factor for cardiovascular death and cardiovascular events but also progression of renal failure in patients with CKD [9, 10].

Another endothelial injury marker is the von Willebrand factor, a glycoprotein produced by endothelial cells and megakaryocytes. vWF is a cofactor of thrombocyte adhesion to connective tissue collagen, thereby exerting prothrombotic action. In the ASCOT study, patients with hypertension (HT) and organ damage (OD) had significantly higher vWF concentrations than those with HT but without OD (137 versus 125 IU/dl). After 6 months of intensive treatment, there was a reduction in systolic blood pressure and a decrease in vWF concentration suggesting an improvement in endothelial function [11].

Increased urinary albumin excretion is not only a symptom of kidney disease but also an exponent of generalized endothelial dysfunction and cardiovascular risk. Because of technical difficulties in obtaining daily urine collection, the albumin to creatinine ratio in the first urine volume is increasingly used in clinical trials. In the study conducted in Denmark by Borch-Johnsen et al., microalbuminuria (>0.65 mg/mmol) significantly increased the risk of ischemic heart disease regardless of other atherosclerosis risk factors [12].

In patients with CKD, both increased reactive oxygen (ROS) and nitrogen radical (RNS) generation and decreased antioxidative potential have been shown [13–15]. Low-density lipoprotein particles modified in the process of oxidation (oxLDLs) develop atherogenic properties and become cytotoxic to vascular endothelial cells, stimulating smooth muscle growth and macrophage attraction. OxLDLs also inhibit macrophage mobility resulting in their accumulation and formation of fatty streaks—the initial stage of the atherosclerotic process [16].

The aim of the study was to assess the association between endothelial dysfunction and oxidative stress biomarkers and cardiovascular risk factors in children with chronic kidney disease.

## 2. Materials and Methods

The prospective study was conducted between June 2008 and February 2011. The study was performed in accordance with the Declaration of Helsinki of 1975 for Human Research and approved by the Bioethical Committee (KBET/17/B/2006). The parents and patients were educated as to the objective and method of performing the study and gave their written informed consent.

### 2.1. Subjects

Patients aged 0–18 years with diagnosed chronic kidney disease and elevated thrombomodulin level (>5 ng/ml) were included.

The exclusion criteria were lack of consent of the patient or parents, congenital heart defects or other primary heart diseases, acute infections during measurements, acute damage, or failure of other organs.

### 2.2. Blood Sampling and Biochemical Analysis

Blood samples for basic research were taken on routine admission for diagnostic check-up in all patients (fasting for 12 hours). Additionally, three samples (for clot, EDTA, and citrate) were collected and centrifuged and plasma and serum were frozen at −80°C. Biochemical analyses were performed, and urea, creatinine, cystatin C, TM, ADMA, brain natriuretic peptide (BNP), vWF, fibrinogen, and lipids were measured. Based on serum creatinine, estimated glomerular filtration rate (eGFR) with the Schwartz et al. [17] and Filler et al. [18] formulas was calculated. Patients were divided into groups depending on CKD stage [group 1: CKD stages 1 + 2 (GFR > 60); group 2: CKD stage 3 (GFR = 30–59); group 3: CKD stage 4 (GFR = 15–29 ml/min/1.73m^2^); group 4: dialyzed children].

To assess endothelial dysfunction and oxidative stress, the concentration of TM, ADMA, vWF, and oxLDL particles and the concentration of carbonyl groups resulting from oxidation of proteins were used. Concentrations of serum thrombomodulin (American Diagnostica Inc., USA), high sensitive C-reactive protein (hsCRP) (R&D Systems, USA), oxLDL (Mercodia Inc., Sweden), protein carbonyl groups (Cayman Chemical Company, USA), intercellular adhesion molecule-1 (ICAM-1) (R&D Systems, USA), and vWF activity (Diagnostica Stago, France) were determined with ELISA. BNP concentrations were measured with the IRMA method (Cis Bio International, France).

### 2.3. Anthropometric and Blood Pressure Measurements

Anthropometric measurements of patients such as weight, height, and waist circumference were measured during each visit, and body mass index (BMI) was calculated. Ambulatory blood pressure measurements (ABPM) with SpaceLabs 90207 device and cuff of appropriate size were performed. Blood pressure (BP) measurements were performed every 20 minutes during the day and every 30 minutes during the night. Mean values of systolic (SBP), diastolic (DBP), and mean BP (mean arterial pressure—MAP) were calculated. Hypertension was defined as BP values equal to or exceeding the 95th percentile for gender, age, and height. BP values were analyzed and expressed in standard deviations (SD).

### 2.4. Echocardiography

Echocardiographic examinations were performed by an experienced cardiologist using HP 5500 unit with S4 and S8 variable frequency probes. In children on chronic hemodialysis, echocardiography was performed on the day between two hemodialysis procedures, while in children on peritoneal dialysis, it was performed during the daily exchange, with a low volume of dialysate in the peritoneal cavity. LV mass (LVM) was calculated by the formula described by Devereux and Reichek [19]. LVM index (LVMI) was obtained by dividing LVM by height^2.7^ to normalize and linearize the relationship between LVM and height [20].

### 2.5. Statistical Analysis

Qualitative values were compared by the chi-square test. Because data of the majority of variables did not show normal distribution, they are presented as median [25th–75th percentile]. Differences between the groups were compared using the Mann–Whitney *U* test. Spearman's rank correlation was used to relate endothelial dysfunction and oxidative stress markers. Statistical calculations were performed using a commercially available statistical package (Statistica PL). A value of *p* < 0.05 was considered significant in all statistical analyses.

## 3. Results

We examined 59 patients (36 boys and 23 girls) aged 0.7 to 18.6 (mean 11.1) years with stages 1 to 5 CKD (7 in stages 1 and 2, 15 in stage 3, 15 in stage 4, and 22 in stage 5) who were under constant medical control in the University Children's Hospital in Krakow. Among diseases leading to the development of CKD in the examined children, the highest prevalence was noted in congenital abnormalities of the kidney and urinary tract (59%) and nephrotic syndrome.

Patients were divided into 2 groups based on the thrombomodulin concentration. A cut-off point of double upper limit of normal (ULN) for elevated thrombomodulin was proposed. Clinical data and basic kidney function parameters depending on thrombomodulin concentration are presented in Table 1.

**Figure 1:**
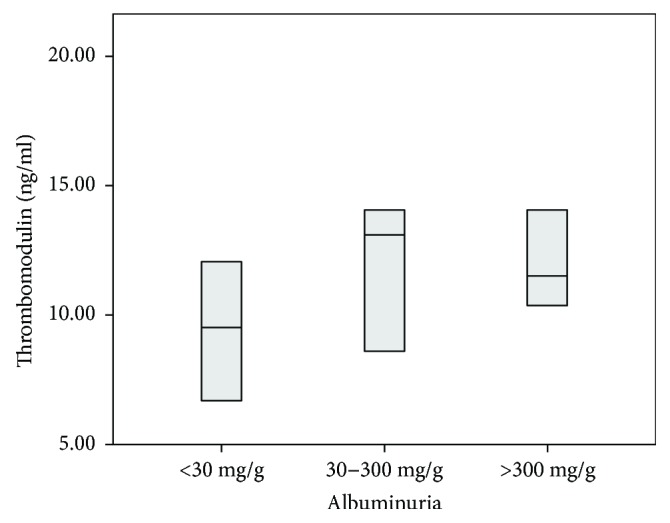
Thrombomodulin concentrations depending on albuminuria groups. Data are presented as median and interquartile range.

There were no significant differences in cholesterol concentrations between children with high and low thrombomodulin (Table 2). Patients with high TM levels had significantly higher ADMA (1.23 (0.90; 1.40) versus 0.94 (0.75; 1.16) *μ*mol/l; *p* < 0.05), BNP (15.63 (5.88; 24.23) versus 4.43 (1.00; 15.39); *p* < 0.05), and oxLDL (88.6 (78.7; 106.9) versus 80.6 (67.4; 91.9); *p* < 0.05) concentrations. The concentrations of vWF, carbonyl groups, hsCRP, ICAM-1, and fibrinogen did not vary significantly (Table 3). Furthermore, they had elevated blood pressure values in 24-hour measurements: systolic (118.0 (113.0; 126) versus 113.0 (105.0; 118.0); *p* = 0.012) and MAP (87.0 (78.0; 92.0) versus 82.0 (77.0; 88.0); *p* = 0.022). There were no differences in other BP values (Table 4). There were no significant differences in thrombomodulin concentrations based on albuminuria groups (see Figure 1).

**Figure 2:**
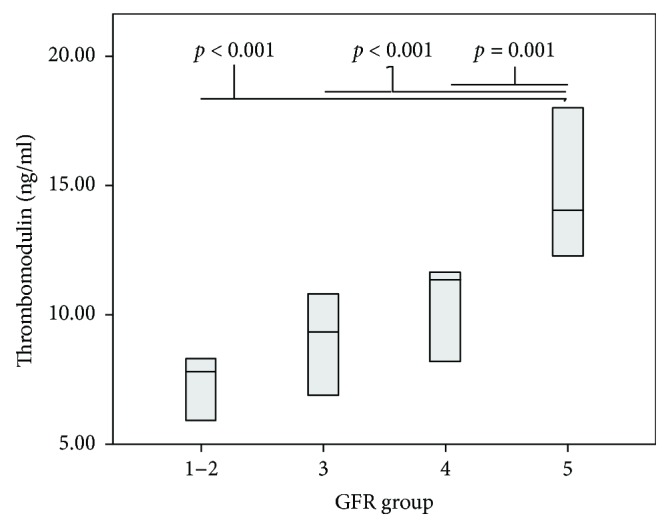
Thrombomodulin concentrations depending on glomerular filtration rate (GFR) group. Group 1-2: CKD stages 1 + 2 (GFR> 60); group 3: CKD stage 3 (GFR = 30–59); group 4: CKD stage 4 (GFR = 15–29 ml/min/1.73m^2^); group 5: dialyzed children. Data are presented as median and intraquartile range.

Thrombomodulin concentrations increased with CKD stage (7.82 versus 9.30 versus 10.44 versus 15.25 ng/ml; *p* < 0.001), respectively, as shown in Figure 2. There were significant differences in median thrombomodulin concentration between CKD stages 1 + 2, 3, 4, and 5 (*p* < 0.001; *p* < 0.001; *p* = 0.003).

**Figure 3:**
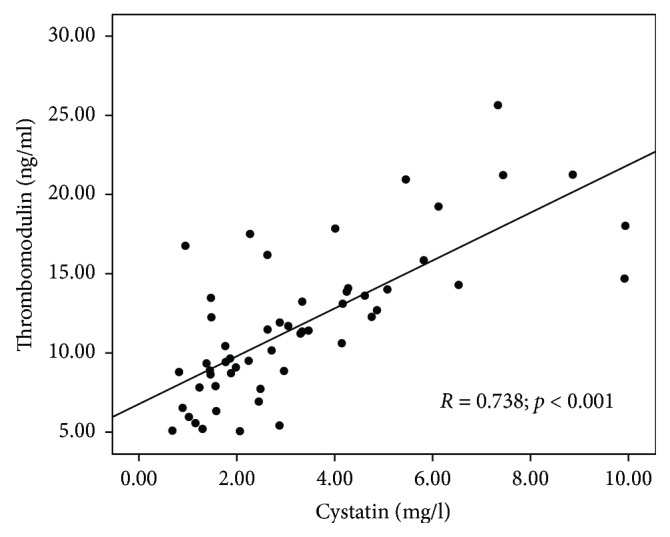
Scatterplot presenting the correlation between cystatin C and thrombomodulin concentrations.

There was a significant correlation between thrombomodulin concentration and markers of renal function—urea (*R* = 0.522; *p* < 0.001), creatinine (*R* = 0.666; *p* < 0.001), and cystatin C (*R* = 0.738; *p* < 0.001) concentrations, eGFR calculated with Schwartz (*R* = −0.677; *p* < 0.001), and Filler formulas (*R* = −0.716; *p* < 0.001). Scatterplot presenting the correlation between cystatin C and thrombomodulin concentrations is shown in Figure 3.

**Figure 4:**
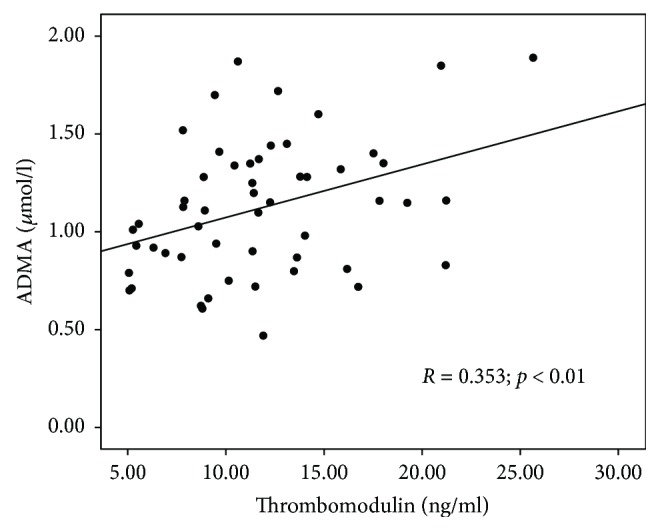
Scatterplot presenting the correlation between asymmetric dimethylarginine (ADMA) and thrombomodulin concentrations.

**Figure 5:**
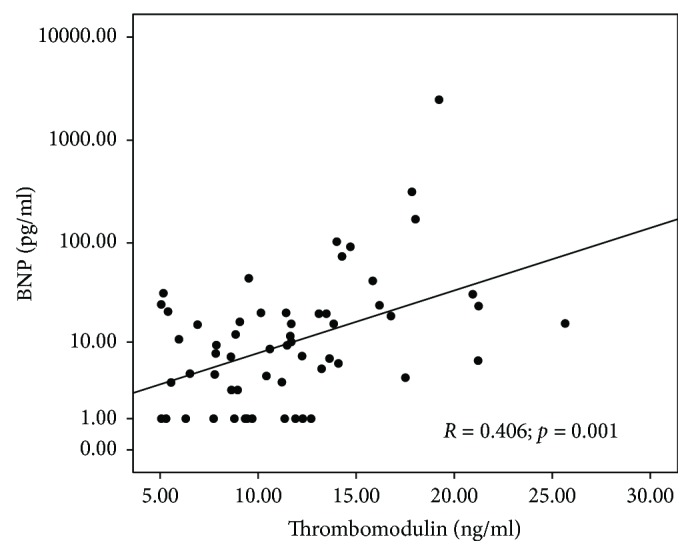
Scatterplot presenting the correlation between brain natriuretic peptide (BNP) and thrombomodulin concentrations.

**Figure 6:**
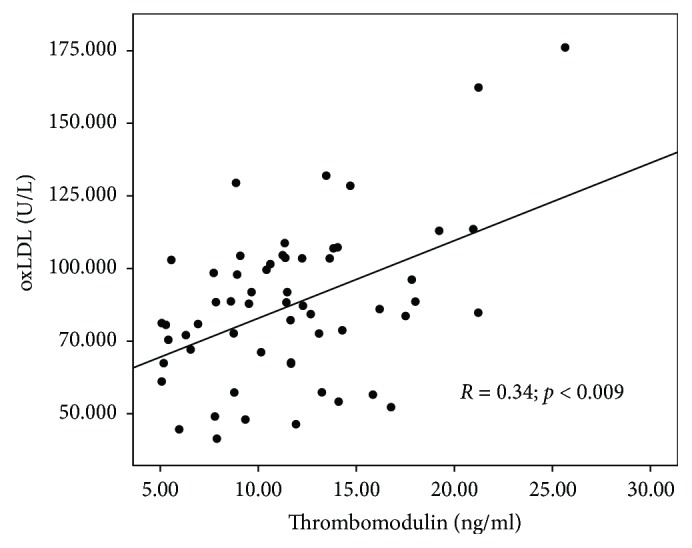
Scatterplot presenting the correlation between oxidized low-density lipoprotein (oxLDL) and thrombomodulin concentrations.

Thrombomodulin concentration correlated significantly with other endothelial dysfunction and oxidative stress parameter concentrations: ADMA (*R* = 0.353; *p* = 0.010), BNP (*R* = 0.406; *p* = 0.001), and oxLDL (*R* = 0.340; *p* = 0.009). Scatterplots presenting the correlations between ADMA, BNP, oxLDL, and thrombomodulin concentrations are shown in Figures 4–6. No correlation between thrombomodulin and vWF (*R* = 0.239; *p* = 0.082), carbonyl groups (*R* = 0.151; *p* = 0.310), hsCRP (*R* = 0.000; *p* = 0.999), and ICAM (*R* = 0.082; *p* = 0.566) concentrations have been found.

**Figure 7:**
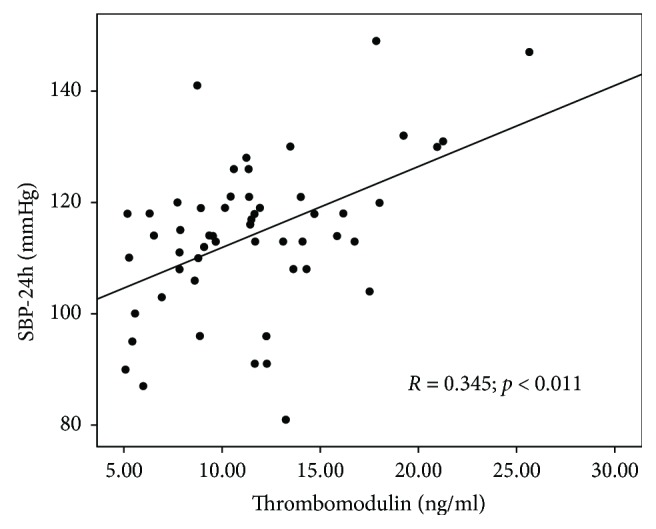
Scatterplot presenting the correlation between mean 24-hour systolic blood pressure (SBP-24h) values and thrombomodulin concentrations.

Thrombomodulin correlated significantly with 24-hour systolic (*R* = 0.345; *p* = 0.011) and mean—MAP (*R* = 0.315; *p* < 0.05) BP values. Scatterplot presenting the correlation between mean 24-hour systolic BP values and thrombomodulin concentrations is shown in Figure 7.

**Figure 8:**
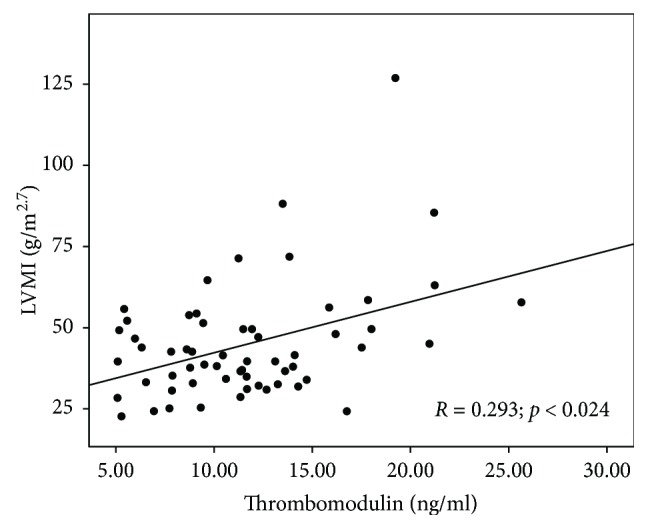
Scatterplot presenting the correlation between left ventricular mass index (LVMI) and thrombomodulin concentrations.

In the studied children with CKD, a significant correlation between thrombomodulin level and LVMI was found (*R* = 0.293; *p* = 0.024). Scatterplot presenting the correlation between LVMI and thrombomodulin concentrations is shown in Figure 8.

## 4. Discussion

This is, to our knowledge, the first study comparing thrombomodulin as endothelial dysfunction marker with oxidative stress markers in children with chronic kidney disease.

In our study, a very strong positive correlation of the concentrations of thrombomodulin and urea, creatinine, and cystatin C and a negative correlation with eGFR were observed, which is evidence of damaging uremic effects on endothelial cells. The highest concentrations of thrombomodulin were observed in dialyzed children. There were also significantly higher renal function parameters and albuminuria in the group of children with thrombomodulin level exceeding over 2 times the upper limit, but there were no differences in cholesterol concentrations.

The CKD population is at increased risk of cardiovascular disease. Until recently, it was considered that this risk was only increased with GFR < 60 ml/min/1.73 m^2^, but modern long-term observations indicate that the increase in cardiovascular mortality is already present with a decrease in GFR below 90 ml/min/1.73 m^2^ [21, 22]. In adult patients with CKD, the cluster of CV risk factors (hypertension, diabetes, hypercholesterolemia, and smoking) is common. Traditional risk factors, however, cannot explain the high prevalence of cardiovascular disease in pediatric population with CKD, as many factors affecting ED such as diabetes, smoking, effect of ageing, long lasting HT as a cause of CKD, and CV comorbidities are not present in this population, which allows for more accurate evaluation of the impact of oxidative stress and uremia on ED.

In the studied group of children with CKD, thrombomodulin correlated well with BNP, a marker of myocardial stress, and LVMI, a marker of left ventricular hypertrophy.

Accelerated atherosclerosis in patients with chronic kidney disease is responsible for increased morbidity and mortality due to cardiovascular causes [23]. Endothelial dysfunction is postulated to be the pathomechanism and early stage of atherosclerosis before the onset of clinically manifested atherosclerotic plaques [24, 25]. Endothelial cell function impairment also plays a role in the progression of atherosclerotic lesions and their clinical complications [26]. Cachofeiro et al. described oxidative stress, endothelial dysfunction, and inflammation as the key triad in the development and progression of atherosclerosis [27]. Thrombomodulin is an anticoagulant cell surface proteoglycan that is cleaved from the endothelial cell surface layer by neutrophil-derived enzymes [28, 29].

In the study by Krzanowski et al., the authors also found a significant correlation between thrombomodulin and serum creatinine, as well as inflammation and endothelial dysfunction markers in CKD. Adult CKD patients with severe radial artery calcification had higher concentrations of TM than patients with less advanced lesions. Thrombomodulin concentrations did not correlate with age and classical cardiovascular risk factors (SCORE) which could indicate that thrombomodulin determines vascular injury and advanced calcification in patients with impaired renal function [30].

The role of thrombomodulin as a vasoprotective agent may also be confirmed by the results of Eguchi et al., who have shown that the supply of recombinant TM reverses FK506-induced ED. Recombinant TM is registered in Japan for DIC treatment [31].

Our study showed a significant correlation between the marker of endothelial dysfunction and a recognized oxidative stress marker—oxLDL. Significant increases in oxLDL levels have been also found in children with markedly increased thrombomodulin levels, which may indicate an intensification of both ED and oxidative stress processes with increasing renal function impairment.

Holvoet and coauthors suggested to measure the concentration of circulating oxidized LDL for a more accurate assessment of cardiovascular risk. Patients with coronary artery disease confirmed in angiography had significantly higher oxLDL levels. There was also a significant correlation between oxLDL and most of the Framingham risk factors [32].

In the studied group, the protein carbonyl content did not increase in the patients with higher thrombomodulin levels. In our previous study, the protein carbonyl content did not increase in advanced stages of CKD [1].

In children, there are currently no studies evaluating TM levels in chronic kidney disease, but endothelial dysfunction has been widely described in nephrotic syndrome. In the study by Tkaczyk et al. and Sharma et al., children with active disease had significantly higher thrombomodulin concentrations compared to control group [33, 34].

In the studied group of children with chronic kidney disease, thrombomodulin correlated significantly with another mediator of endothelial injury—ADMA. ADMA meets multiple uremic toxin criteria—it is a product of protein metabolism, is accumulating in renal failure, is removed during dialysis, and has a specific mechanism of action. In kidney disease, not only ADMA excretion in the urine but also its metabolism (by enzymatic decomposition—dimethylarginine dimethylaminohydrolase) in kidney tissue is reduced.

In the group of 600 patients with long-term type 1 diabetes (mean duration of 28 years), statistically significant differences in ADMA between patients with diabetic nephropathy and normoalbuminuria (0.46 versus 0.4 *μ*mol/l) were found [35]. ADMA concentration was also significantly higher in patients with nephropathy and history of stroke or myocardial infarction compared to patients without cardiovascular events (0.48 versus 0.46 *μ*mol/l). The authors emphasized the fact that ADMA increased in the early stage of diabetic renal disease, that is, at GFR < 76 ml/min/1.73 m^2^.

In the study by Kielstein and coworkers, ADMA was elevated at an early stage of renal failure [36]. Further research is needed to clarify whether elevated ADMA levels in patients with CKD are markers of renal failure or cardiovascular disease mediators [37]. The Zoccali group demonstrated a relationship between ADMA concentration and intima-media thickness in hemodialysis patients. There was also a strong relation between inflammation (CRP), endothelial dysfunction markers (ADMA), and the effect of these factors on the progression of vascular changes in patients with baseline normal IMT [38].

In the group of children with thrombomodulin level exceeding over 2 times the upper limit, significantly higher ADMA levels have been found.

In the studied children with higher thrombomodulin concentrations, significantly higher albuminuria has been found. Albuminuria is another indicator of endothelial injury. The relationship between renal albumin loss and cardiovascular risk in different groups of patients with increased vascular damage (hypertension, diabetes) has been demonstrated. Albuminuria is a recognized cardiovascular risk factor and an indicator of renal failure [39]. In the RENAAL study in subjects with type 2 diabetes, reduction of albuminuria during 6 months of losartan treatment was the strongest factor of cardiovascular protection [40]. An increase in albuminuria by 1 g/g of creatinine in the baseline examination was associated with a 17% increase of cardiovascular endpoint risk and a 26% higher risk of developing heart failure. The Heart Outcome Prevention Evaluation (HOPE) study has shown that even a slight degree of albuminuria increases the risk of cardiovascular events. Each increase in urinary albumin to creatinine ratio by 0.4 mg/mmol increased risk by 5.9% [41]. The association between albuminuria and mortality was also demonstrated in a population of healthy individuals without diabetes and hypertension [42].

The acquired results—significantly higher systolic and mean blood pressure values in patients with higher thrombomodulin levels—prove the well-known influence of hypertension on endothelial damage.

Endothelial dysfunction has been proposed as one of the main mechanisms contributing to the association of chronic kidney and cardiovascular disease. In CKD oxidative stress, subclinical chronic inflammation and uremic toxins are responsible for endothelial cell injury [43]. The potential pathomechanism of increased shedding of TM is the increased cyclic strain caused by hypertension [6].

The lack of correlation between TM and other ED biomarkers (vWF, ICAM-1, and fibrinogen) may be the result of the small study group.

## 5. Conclusions

In children with chronic kidney disease, thrombomodulin seems to be a valuable marker of endothelial dysfunction, correlating strongly with CKD stage, kidney function parameters (urea, creatinine, and cystatin C), as well as oxidative stress, hypertension, and left ventricular hypertrophy.

## Figures and Tables

**Table 1:** Basic clinical data and kidney function parameters depending on thrombomodulin concertation in the investigated group of 59 patients.

Parameter	Thrombomodulin		*p* value∗
	<2xULN (*n* = 26)	≥2xULN (*n* = 33)	
Age (years)	10.5 (5.0; 16.0)	12.1 (9.1; 15.3)	0.277
Male sex (*n*)	17 (65.4%)	19 (57.5%)	0.599
Height (z-score)	−0.35 (−1.77; 0.07)	−1.34 (−2.38; −0.27)	0.097
Body mass (z-score)	−0.84 (1.30; 0.11)	−1.06 (−2.55; −0.01)	0.320
BMI (kg/m^2^)	16.5 (15.8; 18.6)	16.8 (14.9; 20.1)	0.970
BMI (z-score)	−0.62 (−1.11; 0.53)	−0.66 (−1.45; 0.44)	0.903
Creatinine (*μ*mol/l)	110.8 (93.8; 179.3)	403.0 (283.3; 725.3)	<0.001
Cystatin (mg/l)	1.58 (1.25; 2.06)	4.21 (3.05; 5.82)	<0.001
eGFR Filler (ml/min/1.73 m^2^)	55.2 (38.8; 72.0)	18.4 (12.0; 26.2)	<0.001
Microalbuminuria (mg/l)	18.0 (10,7; 63.0)	99.5 (24.5; 423.0)	0.005

Values presented as median (25th–75th percentile). ^∗^Mann–Whitney *U* test. ULN: upper limit of normal; BMI: body mass index; eGFR: estimated glomerular filtration rate.

**Table 2:** Cholesterol concentrations depending on thrombomodulin concentration in the investigated group of 59 patients.

Parameter	Thrombomodulin		*p* value∗
	<2xULN (*n* = 26)	≥2xULN (*n* = 33)	
Total cholesterol (mmol/l)	4.62 (4.13; 5.12)	4.96 (4.15; 6.04)	0.465
TGL (mmol/l)	1.47 (1.23; 2.09)	1.60 (1.25; 2.82)	0.769
HDL (mmol/l)	1.27 (1.12; 1.56)	1.19 (1.03; 1.61)	0.500
LDL (mmol/l)	2.59 (2.30; 2.91)	2.31 (2.01; 3.29)	0.975

Values presented as median (25th–75th percentile). ^∗^Mann–Whitney *U* test. ULN: upper limit of normal; TGL: triglycerides; HDL: high-density lipoprotein; LDL: low-density lipoprotein.

**Table 3:** Oxidative stress and endothelial dysfunction parameters depending on thrombomodulin concentration in the investigated group of 59 patients.

Parameter	Thrombomodulin		*p* value∗
	<2xULN (*n* = 26)	≥2xULN (*n* = 33)	
vWF (%)	87.8 (78.8; 93.3)	90.2 (82.8; 96.7)	0.521
Carbonyl groups (nmol/mg)	1.18 (0.61; 1.89)	1.36 (0.82; 2.17)	0.634
ADMA (*μ*mol/l)	0.94 (0.75; 1.16)	1.23 (0.90; 1.40)	0.029
BNP (pg/ml)	4.43 (1.00; 15.39)	15.63 (5.88; 24.23)	0.021
oxLDL (U/l)	80.6 (67.4; 91.9)	88.6 (78.7; 106.9)	0.033
hsCRP (ng/ml)	373.2 (132.6; 736.1)	310.7 (145.6; 878.1)	0.535
ICAM-1 (ng/ml)	373.2 (132.6; 736.1)	323.6 (290.3; 384.4)	0.977
Fibrinogen (g/l)	3.5 (2.5; 4.4)	3.3 (2.4; 4.4)	0.856

Values presented as median (25th–75th percentile). ^∗^Mann–Whitney *U* test. ULN: upper limit of normal; vWF: von Willebrand factor; ADMA: asymmetric dimethylarginine; BNP: brain natriuretic peptide; oxLDL: oxidized low-density lipoprotein; hsCRP: high sensitive C-reactive protein; ICAM-1: intercellular adhesion molecule-1.

**Table 4:** 24-hour blood pressure values depending on thrombomodulin concentration in the investigated group of 59 patients.

Parameter	Thrombomodulin		*p* value∗
	<2xULN (*n* = 26)	≥2xULN (*n* = 33)	
24 h SBP (mmHg)	113.0 (105.0; 118.0)	118.0 (113.0; 126)	0.012
24 h DBP (mmHg)	65.0 (61.0; 75.0)	69.0 (62.0; 78.0)	0.063
24 h MAP (mmHg)	82.0 (77.0; 88.0)	87.0 (78.0; 92.0)	0.022
24 h SBP (SD)	0.242 (−1.408; 0.746)	0.805 (−0.875; 2.337)	0.119
24 h DBP (SD)	−0.109 (−1.403; 1.375)	0.956 (−1.025; 3.511)	0.103
24 h MAP (SD)	0.309 (−0.858; 1.040)	1.472 (−0.646; 3.145)	0.076

Values presented as median (25th–75th percentile). ^∗^Mann–Whitney *U* test. ULN: upper limit of normal; SBP: systolic blood pressure; DBP: diastolic blood pressure; MAP: mean arterial pressure.
